# Transcriptomic analysis of shrimp immune modulation by dietary inactivated microbial cells and *Sargassum* sp. immunostimulants

**DOI:** 10.1016/j.cirep.2026.200283

**Published:** 2026-04-19

**Authors:** M.A. Amatul-Samahah, F.M.I. Natrah, M.N.A. Amal, M.Y. Ina-Salwany

**Affiliations:** a Department of Aquaculture, Faculty of Agriculture, Universiti Putra Malaysia, 43400, Serdang, Selangor, Malaysia; b Aquatic Animal Health and Therapeutics Laboratory (AquaHealth), Institute of Bioscience, Universiti Putra Malaysia, 43400 Serdang, Selangor, Malaysia; c Institute of Bioscience, Universiti Putra Malaysia, 43400, Serdang, Selangor, Malaysia; d Fisheries Research Institute Glami Lemi, Titi, 71650, Jelebu, Negeri Sembilan, Malaysia

**Keywords:** AHPND, Immunostimulantion, *Penaeus vannamei*, Shrimp immune, Transcriptome

## Abstract

Research on the shrimp immune system has piqued attention in recent years, as a better understanding of the events that occur in the shrimp immune system could assist to improve shrimp's overall health and, as a result, increase shrimp production output. This study focuses on *Litopenaeus vannamei,* employing transcriptomic analysis to understand gene expression and immune responses. Shrimp were immunostimulated using feed-based inactivated microbial cells with *Sargassum* sp.. Shrimp treated with immunostimulants exhibited no physical changes after immunisation, and histological investigation of the hepatopancreas indicated normal cell architectures across groups given 1 × 10^5^ CFU kg/feed + Commercial feed with 2% *Sargassum* sp. via oral route. RNA sequencing and quality assessment were performed, resulting in 84,545 transcripts and 47,763 unigenes. Functional annotation using multiple databases (NR, NT, KO, SwissProt, PFAM, GO, KOG) revealed that 63.36% of unigenes could be annotated, with significant matches to known immune-related genes. Differential expression analysis identified 2868 up-regulated and 4721 down-regulated genes between the treated and control groups. Key immune-related genes, including those involved in pathogen response, antimicrobial peptide production, and various immune signaling pathways (e.g., Toll, JAK-STAT, P13K-AKT, Wnt), showed significant differential expression. This study provides valuable insights into the molecular basis of shrimp immune responses, highlighting the potential of functional genomics to enhance shrimp health and production through targeted immunostimulants.

## Introduction

Immunostimulation in shrimp, particularly in species like *Litopenaeus vannamei* has become a crucial area of research due to the significant economic value of shrimp farming and the challenges posed by disease outbreaks. Traditional disease control methods involving antibiotics and chemical disinfectants are being phased out due to concerns about bacterial resistance and environmental impact [1]. The use of immunostimulants derived from natural sources such as seaweed, spirulina, and plant extracts has emerged as a promising alternative to enhance the non-specific immune response in shrimp, thereby improving disease resistance and overall production in aquaculture [2]. This seaweed is rich in bioactive compounds such as polysaccharides, polyphenols, and antioxidants, which are known to boost immune responses in aquatic animals. Combining Sargassum sp. with inactivated bacteria was hypothesized to synergistically enhance shrimp immunity by leveraging the immunostimulatory properties of both components [2]. These natural immunostimulants can be administered through various methods like oral intake, immersion, or injection, and have shown to boost parameters like total hemocyte count, phagocytosis activity, and phenoloxidase activity in shrimp, contributing to a more sustainable and eco-friendly approach to disease control in shrimp farming [3].

Transcriptome analysis plays a crucial role in understanding the immune responses of shrimp, particularly in the context of Acute Hepatopancreas Necrosis Disease (AHPND) caused by *Vibrio parahaemolyticus* [[4], [5], [6], [7], [8]]. Studies have shown that the use of immunostimulants, such as natural products like *Pandanus tectorius* leaf extract, can enhance the immune response of white-leg shrimp (L. *vannamei*) against pathogens like *V. parahaemolyticus,* improving survival rates and upregulating immune-related genes like Hsp70 and crustin [9]. By analyzing the RNA transcripts present in different tissue samples of shrimp, researchers can gain insight into which genes are actively being expressed and how they may be related to specific traits [10,11]. It is also an interesting method of obtaining insights into the molecular basis of immune reactions in this species where little research has been done.

In shrimp studies, expressed sequence tags (ESTs) analysis has helped to converge knowledge on genes with similarity to known immune function genes from other organisms (such as protease inhibitors) that can respond to immune stimulation in shrimp [12,13]. The large-scale EST and high-throughput gene expression studies will increase the likelihood that sound hypotheses can be formulated regarding the roles of candidate immune function genes in shrimp. Besides that, the analysis has revealed that a high proportion of ESTs from shrimp share no significant similarity to any known sequences [14,15]. Furthermore, the identification of immune-related genes in L. *vannamei* sheds light on the molecular mechanisms underlying shrimp immunity and response to pathogens, highlighting the importance of understanding the host metabolism in combating infections like AHPND [16]. Substantial insights have been gained in recent years into important aspects of the crustacean immune system, including the role of phagocytic cells, the prophenoloxidase cascade, melanization system, and antimicrobial peptides [[17], [18], [19], [20]]. While some of these well-conserved immune effector pathways (such as melanization and antimicrobial peptide production) are reasonably well understood at the biochemical level, the molecular events that underlie the majority of crustaceans such as in shrimp immune reactions remain unknown. Some of the greatest gaps in our knowledge of crustacean immunity concern the molecular basis for its response toward immunostimulation.

Thus, in this study, we wanted to understand the effect of immunostimulation in shrimp using feed-based inactivated microbial immunostimulant with prebiotic, *Sargassum* sp., and its effect on shrimp immune system at the transcriptional level. Additionally, we also would like to elucidate the pathways involving immune-related genes in treated samples.

## Materials and methods

### Ethics statement

This research was carried out in strict accordance with the recommendations for the use of animals regulated by the Institutional Animal Care & Use Committee Universiti Putra Malaysia (IACUC), UPM. Shrimp handling and experimental protocols in this study were approved by the ethics committee at Universiti Putra Malaysia, UPM (20190416151439AUP/101).

### Preparation of feed-based inactivated microbial cells with Sargassum sp

Inactivated microbial cell was prepared using *Vibrio parahaemolyticus* C4B strain as per the method described previously [21]. The experiment was carried out in Biotechnology Aquatic Laboratory, Department of Aquaculture, Faculty of Agriculture, Universiti Putra Malaysia. The *V. parahaemolyticus* C4B strain selected as the bacterial strain for preparation of the inactivated microbial immunostimulant was due to the strain association with AHPND disesase and has been characterized previously [22]. The *V. parahaemolyticus* C4B strain was cultured in saline tryptic soy broth (STSB) with 1.5% NaCl wt/vol for 24 to 36 h at room temperature. The bacterial cells were harvested by centrifugation at 13,500 g**.** The sediment cells were re-suspended in sterile PBS**,** and heat-inactivated at 70 °C for 30 min [23]. The complete inactivation of *V. parahaemolyticus* was confirmed by culturing heat-treated bacterial suspensions on saline tryptic soy agar plates. No bacterial growth was observed after 48 h of incubation. A cell density of 10^9^ CFU/mL of the inactivated bacteria corresponding to an OD of 0.85 at 575 nm was used for diluting or concentrating the bacterial suspension. Meanwhile, the experimental feed (Cargill, Malaysia commercial pellet: 45%) was incorporated with *Sargassum* sp. was at 2% by using method as stated by Nazaruddin et al. [24].

For the preparation of experimental diet, the inactivated microbial cell suspension was prepared by diluting the suspension in water to obtain the final concentration of 10^5^ CFU kg/feed. The suspension was mixed with 0.1% guar gum (Merck, Germany) as binder and applied uniformly on the feed pellet previously replletized with 2% *Sargaasum* sp. The control diet used in this experiment is Cargill, Malaysia commercial pellet (No.2, 45%).

### Shrimp and feed-based immunostimulant treatment

A total of healthy 500 *P vannamei* post-larvae (PL15) with uniform-sized (0.24 ± 0.01 g) were used in this study. The shrimp were supplied by a commercial fish farmer (Oasis Long Diann Bio-Tech Sdn Bhd, Banting, Selangor Darul Ehsan, Malaysia). The experiments were done at the Hatchery Unit, Institute of Bioscience (IBS), Universiti Putra Malaysia (UPM). Upon arrival, the shrimp was immersed in 500 L fiberglass tanks that were filled with aerated seawater for 30 min before separated into few tanks for acclimatization procedures [25]. Shrimp were maintained in closed recirculating tanks with constant aeration and fed twice a day with crumbled commercial shrimp feed (Cargill, Malaysia). Water temperature in the experimental tanks was set at 28 °C. The seawater was aerated using the Resun air pump LP100 (Shenzhen Xing Risheng Industrial Co. Ltd, China) that was connected by a clear tube attached with air stone to each of an operated tank and aquarium. The salinity of the seawater was maintained between 20 and 26 ppt.

The immunostimulant administration strategies were carried out for four weeks for (n = 40 shrimp PL in each treatment group). The immunostimulants were fed to shrimp post-larvae through oral (feeding rate at 5% of shrimp total body weight). Feeding was provided twice daily, one session in the early morning and the second session was in the late afternoon [23]. The control group were fed with commercial pellet (Cargill, Malaysia, No.2, 45%) with the same feeding rate (Table 1). A consistent feeding schedule was found to reduce cannibalisms. About 10% of the seawater tank volume was changed daily one hour after second feeding session. The left out sinking feed were discarded manually using syphon tube and a small net. The water quality parameters in the tanks were maintained within acceptable limits for shrimp culture.

**Table 1:** The treatment groups of shrimp sample for transcriptome analysis.

Treatments	Administration method	Dosage
Commercial feed	Oral	-
1 × 10^5^ CFU kg/feed + Commercial feed with 2% *Sargassum* sp.	Oral	1 × 10^5^ CFU kg/feed

### Histopathology

Histological analysis was performed to study on the pathologic changes at the histological level. The samples were taken whole body parts of shrimp including, head, tail, hepatopancreas, muscle tissue, and exoskeleton. All the samples were fixed in 10% of formalin (v/v). Histology analysis was conducted in Veterinary Diagnostics Laboratory, Department of Laboratory Diagnostics, Faculty of Veterinary Medicine, Universiti Putra Malaysia (UPM), Malaysia, following the routine protocols for preparation of tissues and paraffin embedding techniques [26]. The paraffin blocks were cut at 4 μm thick paraffin sections and stained with haematoxylin and eosin (H&E). The slides were examined under a light microscopy by using Axioskop 2 (Carl Zeiss Jena GmbH, Germany), attached with AxioCamERc5s microscope cameras (Carl Zeiss Jena GmbH, Germany). The microscope was also installed to ZEN 2.3 Lite software platform in order to control the camera and to export czi format images to jpeg format.

### RNA extraction and quality evaluation

Shrimp samples obtained from the control group and treated with immunostimulants were stored in RNAlater solutions (Thermofisher Scientific, USA). Total RNA extraction from shrimp samples was performed using the TRIzol RNA isolation (Invitrogen, USA) protocol [27]. An on-column DNase treatment was performed during RNA purification to remove residual gDNA. RNA degradation and contamination were measured using 1% agarose gels. The RNA purity was determined using the NanoPhotometer® spectrophotometer (IMPLEN, CA, USA). RNA Integrity Number (RIN) and quantitation were assessed using the RNA Nano 6000 Assay Kit of the Agilent Bioanalyzer 2100 system (Agilent Technologies, CA, USA).

### Library construct and sequencing

A total amount of 1 μg of RNA per sample was used as input material for the RNA sample preparations. Sequencing libraries were generated using NEBNext® Ultra™ RNA Library Prep Kit for Illumina® (NEB, USA) following the manufacturer’s recommendations and index codes were added to attribute sequences to each sample. Briefly, mRNA was purified from total RNA using poly-T oligo-attached magnetic beads. Fragmentation was carried out using divalent cations under elevated temperature in NEBNext First Strand Synthesis Reaction Buffer (5X).

First strand cDNA was synthesized using random hexamer primer and M-MuLV Reverse Transcriptase (RNase H-). Second strand cDNA synthesis was subsequently performed using DNA Polymerase I and RNase H. Remaining overhangs were converted into blunt ends via exonuclease/polymerase activities. After adenylation of 3‟ ends of DNA fragments, NEBNext Adaptor with hairpin loop structure was ligated to prepare for hybridization. In order to select cDNA fragments of preferentially 250∼300 bp in length, the library fragments were purified with AMPure XP system (Beckman Coulter, Beverly, USA). Then 3 μl USER Enzyme (NEB, USA) was used with size-selected, adaptor-ligated cDNA at 37 °C for 15 min followed by 5 min at 95 °C before PCR. Then PCR was performed with Phusion High-Fidelity DNA polymerase, Universal PCR primers and Index (X) Primer. At last, PCR products were purified (AMPure XP system) and library quality was assessed on the Agilent Bioanalyzer 2100 system.

### Quality control of sequencing

Data file from high-throughput sequencing was transformed into sequenced reads by CASAVA 1.6 base recognition (base calling). Raw data were stored in FASTQ (fq) format files, which contain sequences of reads and corresponding base quality. The data quality control was carried out by analyzing the error rate, GC content distribution and data filtering. Sequencing error rate and base quality varied depending on sequencers, reagent residues, and different sample types. For RNA-seq technology, the error rate increased with the sequencing reads for the consumption of sequencing reagents [28]. GC content distribution analysis was to detect potential AT/GC separation, which affects subsequent gene expression quantification.

Raw data (raw reads) of fastq format were first processed through in-house per scripts. In this step, clean data (clean reads) were obtained by removing reads containing adapter, reads containing ploy-N and low quality reads from raw data. At the same time, Q20, Q30, GC-content and sequence duplication levels of the clean data were calculated. The sequencing quality score of a given base, Q, is defined by the following equation: Q = −10log_10_(e), where e is the estimated probability of the base call being wrong. A quality score of 20 (Q20) represents error rate of 1 in 100, with a corresponding call accuracy of 99%. Similarly, Q30 means the call accuracy of 99.9%. All the downstream analyses were based on clean data with high quality.

### De novo assembly and functional annotation of unigenes

Clean reads were de novo assembled using Trinity, a professional transcriptome assembler comprising three components: Inchworm, Chrysalis, and Butterfly. Inchworm assembles reads into linear contigs by searching for paths in a k-mer graph. Chrysalis then pools these contigs if they share k-1-mers and builds individual de Bruijn graphs. Butterfly processes these graphs by trimming spurious edges, compacting linear paths, and reconciling them with reads to produce linear sequences for each splice form and paralogous transcript [29]. Clustering was performed using Corset 1.0, which clusters contigs based on shared reads and expression patterns, selects the longest transcripts as unigenes, and filters out contigs with fewer than ten mapped reads [30]. BUSCO v5.4.4 evaluated the integrity of the transcripts by analyzing the splicing results and assessing the quality and accuracy of the splicing based on single-copy orthologs [31].

For annotation analysis, seven databases were used NR, NT, Pfam, COG/KOG, Swiss-Prot, KEGG, and GO. NR and NT are formal NCBI databases for protein and nucleotide sequences, respectively, including information from various repositories such as GenBank and SwissProt. For the NCBI NT database, the software and parameters used were NCBI BLAST (Basic Local Alignment Search Tool) 2.13.0+ (https://blast.ncbi.nlm.nih.gov/Blast.cgi) and e value threshold was 1e-5. For NCBI NR, SwissProt and KOG, DIAMOND v0.8.31 [32] was used and the cut-off e-value threshold was 1e-5. For PFAM (the prediction of protein structure domain), HMMER version 3.0 (http://hmmer.org/) [33] and the e value threshold was 0.01. For GO (based on the protein annotation results of NR and Pfam), Blast2GO (https://www.blast2go.com/) [34] was used and the e-value threshold was 1e-6. For KEGG, KAAS (KEGG Automatic Annotation Server) (https://www.genome.jp/kegg/kaas/) [35] was used with the e value threshold which was 1e-5.

### Identification of differentially expressed genes (DEGs)

The input data for differential gene expression analysis were read counts from gene expression level analysis. The differential gene expression analysis contains three steps which were first read counts normalization, second step model dependent p-value estimation and the third step, False Discovery Rate (FDR) value estimation based on multiple hypothesis testing. The FDR, is defined as the expected fraction of false rejections among those hypotheses rejected. Different softwares and parameter sets are applied in different situations. Differential expression analysis of two groups was performed using the DESeq R package (1.10.1) (https://bioconductor.org/packages/release/bioc/html/DESeq2.html) [36]. DESeq provides statistical analysis for determining differential expression in digital gene expression data using a model based on the negative binomial distribution. The resulting P values were adjusted using the Benjamini-Hochberg method [36,37] for controlling the FDR. Genes with an adjusted P-value < 0.05 found by DESeq were assigned as differentially expressed.

### GO enrichment analysis and KEGG pathway enrichment analysis

Gene Ontology (GO) enrichment analysis done to determined any statistically significant over-representations of GO terms within a set of genes or proteins. GO terms (http://geneontology.org/docs/go-enrichment-analysis/) [38] with padj (adjusted p-value) <0.05 were significant enrichment. The three different classifications represented the three basic classifications of GO terms were biological processes (BP), cellular components (CC), and molecular functions (MF). GO classification correlated the gene with its GO function to get gene annotation information. GO enrichment examined the enrichment of a group gene with a similar GO function, which was used to analyze the gene with similar function. DAG (Directed Acyclic Graph) was used visually to display the enriched GO term of differential expression genes and their hierarchy. KEGG (Kyoto Encyclopedia of Genes and Genomes) pathway enrichment analysis (https://www.genome.jp/kegg/pathway.html) studied the interactions of multiple genes that may be involved in certain biological functions [39]. The pathway enrichment analysis identified significantly enriched metabolic pathways or signal transduction pathways associated with differentially expressed genes compared with the whole genome background. KOBAS software was used to test the statistical enrichment of differential expression genes in KEGG pathways [38].

### Analysis of immune-related genes and signaling pathways in shrimp

Based on the differential gene expression analysis, immune-related genes were identified and the signaling pathways involved were determined. The immune-related genes with log_2_(Fold Change)>1 value were considered differentially expressed and were statistically significant based on padj<0.05 (with biological replicates). The pathways involved in the immune-related genes and signalling pathways were also identified.

### Statistical analysis

The significance fold change of the means values between the experimental and control groups was analyzed by one-way analysis of variance (ANOVA). Duncan’s new multiple range test was applied for data comparison using IBM SPSS statistics software version 22 (SPSS Inc., Chicago, IL, USA), and differences at P < 0.05 were considered significant.

## Results

### Effects of feed-based immunostimulant on general and histomorphology of hepatopancreas

**Fig. 1:**
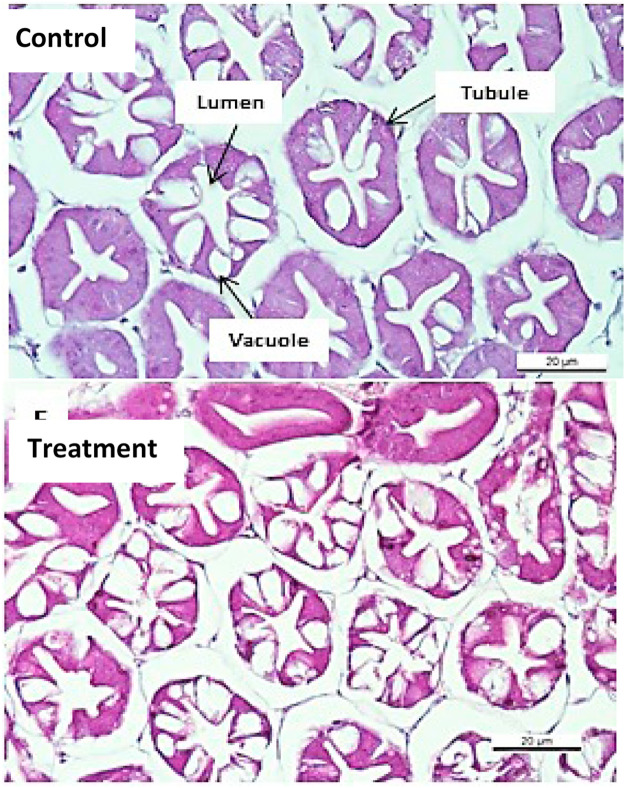
Hepatopancreas of L. *vannamei* immunized with immunostimulant 1 × 10^5^ CFU kg/feed with 2% *Sargassum* sp. Shrimp fed with commercial diet was labelled as control. Slides were viewed under microscope. Scale bars: 20 µm.

Following immunization after four weeks, all shrimp did not show any physical and appeared normal without gross lesions or physical changes. Physical changes or infections including lethargy, erratic swimming behaviour, reduced feed intake, off-white body colour, and anorexia which are consistent in AHPND infections were not observed. Based on the histomorphological analysis, the hepatopancreatic cells and structures were normal and remained unchanged by the immunization of the immunostimulants (Fig. 1). Histopathological features such as sloughing of epithelial tubule, hemocytic infliltration, bacterial colonization, karyomegaly and white feces syndrome which were expected of AHPND infection shrimp were not observed in the shrimp studied.

### Illumina sequencing, quality assessment and de novo assembly

In order to further confirm the expression of an immunological response after immunostimulation, the total RNA from the immunized shrimp and control shrimp in duplicate samples were subjected to sequencing. Clean reads are de novo assembled by Trinity to get assembly transcriptome as in *de novo* analysis where no reference genome is available, all readings from the samples were assembly together in order to obtained the reference genome. A total of 84,545 transcripts were obtained together and 47,763 unigenes were obtained Table 2.

**Table 2:** Overview of the number of transcripts and unigenes in different length intervals.

Transcript length interval	300–500bp	500–1kbp	1k-2kbp	>2kbp	Total
Number of transcripts	27,906	20,894	16,881	18,864	84,545
Number of Unigenes	20,101	12,816	7830	7016	47,763

Based on the statistical summary of reads obtained, Library G1_A produced 38,465,352 clean reads, Library G1_B produced 35,324,298 clean reads, Library G6_A produced 31,049,285 clean reads and Library G6_B produced 30,313,061 clean reads (Table 3). For Q20 which was the percentage of the bases whose Q Phred values are greater than 20 (Number of bases with Q Phred value > 20) / (Number of total bases) *100), all clean reads from each library showed a value of more than 97%. Meanwhile, for Q30 which was the percentage of the bases whose Q Phred values are greater than 20 (Number of bases with Q Phred value > 30) / (Number of total bases) *100), all clean reads from each library showed value more than 93% (Table 3). Higher Q scores indicated a smaller probability of error and lower Q scores can result in a significant portion of the reads being unusable leading to increased false-positive variant calls, resulting in inaccurate conclusions. Quality score, Q30 is considered a benchmark for quality in next-generation sequencing (NGS) as the probability of incorrect base call is just 1 in 1000. Based on Table 3, Guanine-Cytosine (GC) content for clean reads of Library G1_A and Library G1_B was close to 46%. The GC content for clean reads of Library G6_A and Library G6_B were closed to 45%. The above result indicated that the sequence data harvested good quality for further analysis.

**Table 3:** Summary of the sequencing data of transcriptomic analysis.

Library ID	Raw reads	Raw bases	Clean reads	Clean bases	Error rate (%)	Q20	Q30	GC (%)
G1_A	38,994,771	11.7	38,465,352	11.5	0.03	97.46	93.15	46.62
G1_B	35,785,588	10.7	35,324,298	10.6	0.03	97.43	93.08	46.42
G6_A	31,425,033	9.4	31,049,285	9.3	0.03	97.25	92.66	45.47
G6_B	30,742,265	9.2	30,313,061	9.1	0.03	97.59	93.41	44.92

### Functional annotation and classification

**Fig. 2:**
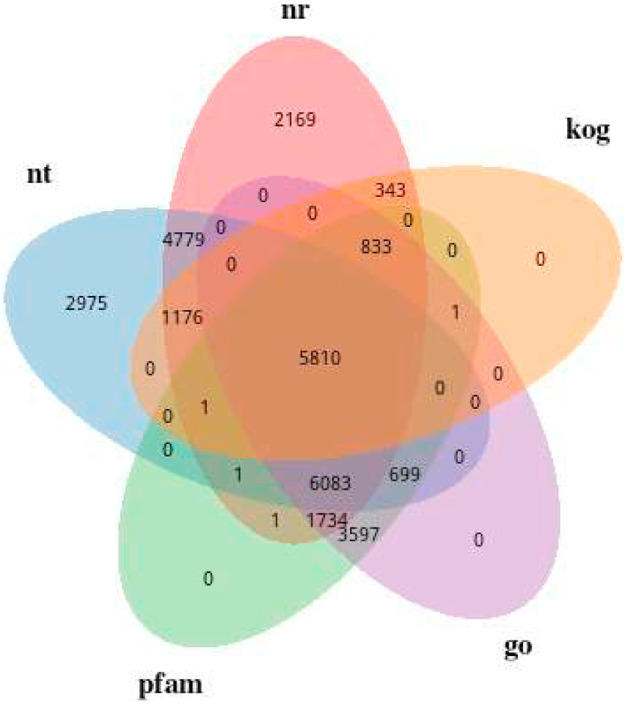
Mapping unigenes using Venn diagram. Overlapping areas show the number of unigenes successfully mapped to the overlapping databases. Results are shown only for the Non-redundant protein database (NR), euKaryotic Orthologous Groups (KOG), Gene ontology (GO) and Nucleotide sequence database (NT), and Protein family (PFAM).

In this study, we have managed to annotate 47,763 (63.36%) of our unigenes using seven databases (Non-Redundant protein database (NR), Nucleotide sequence database (NT), Gene ontology (GO), euKaryotic Orthologous Groups (KOG), KEGG Orthology (KO), SwissProt, and Protein family (PFAM) (Table 4). Based on the annotation results, 22,930 of our unigenes (48.00%) had similar matches in NR database, while 21,524 (45.06%) were significantly similar in nucleotide sequence (NT) database. The unigenes were independently annotated in the KO, SwissProt, PFAM, GO and KOG databases. The percentage of genes that were successfully annotated in each functional database was 20.03%, 30.99%, 39.27%, 39.27% and 17.09% in the KO, SwissProt, PFAM, GO and KOG databases respectively. Fig. 2 depicted the Venn diagram of unigene mapping results and areas of overlap showed the total number of unigenes able to be mapped to the overlapping databases. The Venn diagram presents the distribution and overlap of annotated sequences across five databases: NR, NT, KOG, GO, and Pfam. A large number of sequences (5810) are shared among all databases, indicating a well-characterized core set with strong agreement in sequence similarity, functional annotation, and conserved domains. This suggests high confidence in the predicted biological roles of these sequences. Additionally, NT (2975) and NR (2169) contain notable numbers of unique annotations, reflecting sequences that are identifiable through similarity searches but lack further classification in functional or domain-based databases.

**Table 4:** Percentage of genes that were successfully annotated in each functional database searched. Overall indicates the number of unigenes that were annotated by at least one functional database.

Annotation	Number of Unigenes	Percentage (%)
Annotated in NR	22,930	48.00
Annotated in NT	21,524	45.06
Annotated in KO	9567	20.03
Annotated in SwissProt	14,803	30.99
Annotated in PFAM	18,760	39.27
Annotated in GO	18,757	39.27
Annotated in KOG	8164	17.09
Annotated in all Databases	4533	9.49
Annotated in at least one Database	30,264	63.36
Total Unigenes	47,763	100.00

**Fig. 3:**
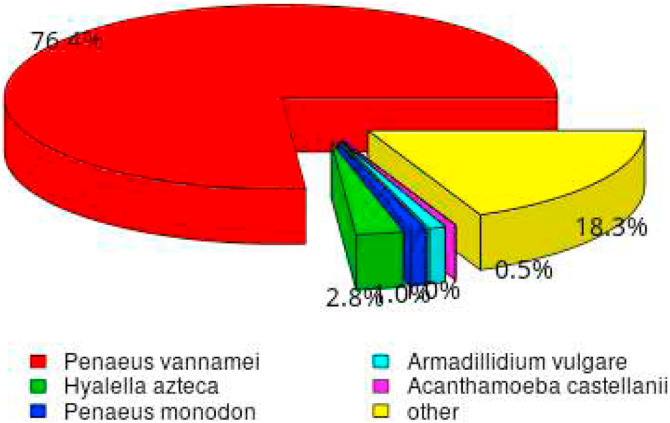
Pie chart is the species distribution similarity (%) of unigenes annotated from NR databases.

Partial overlaps between databases, such as between NR and NT or among Pfam, GO, and KOG, indicate sequences with intermediate levels of annotation. These may have identifiable domains or partial functional information but are not fully characterized across all databases. Overall, the results highlight that sequence-based databases (NR and NT) provide broader coverage, while functional databases (GO, KOG, Pfam) offer more specific but limited annotations. Combining multiple databases therefore improves the overall reliability and depth of functional interpretation. Fig. 3 showed a percentage similarity (%) species distribution of unigenes annotated from non-redundant protein database (NR) database with the species studied which is *P. vannamei.* Based on the unigenes annotation in NR databases, the unigenes were significantly similar *P. vannamei* (76.4%).

### GO, KOG, and KEGG classification of transcriptome sequences

**Fig. 4:**
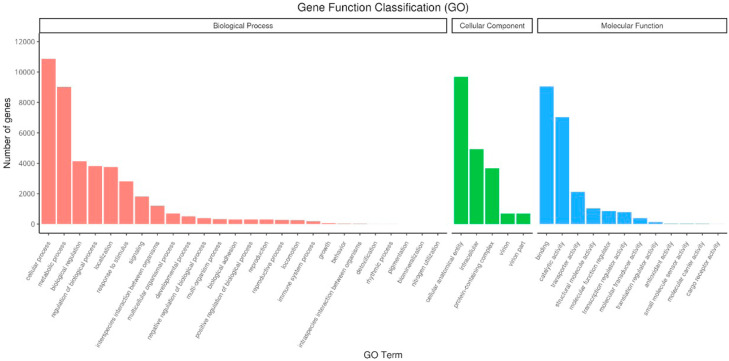
Gene Ontology classifications of the assembled unigenes (GO). The results were categorized into three major categories: cellular component, molecular function, and biological process. The right y-axis indicated the number of genes in a category. The left y-axis indicated the percentage of a specific category of genes in that main category.

Gene Ontology annotation effectively annotated genes categorized into three GO domains namely cell component (CC), biological process (BP), and molecular function (MF) (Fig. 4). Biological process genes roughly convoluted about 26 numbers of genes but were mainly involved in cellular process (10,865 genes), metabolic process (9013 genes), biological regulation (4162 genes), regulation of biological process (3818 genes), localization (3750 genes) response to stimulus (2813 genes) and signaling (1802 genes). 166 genes were classified under the immune system process (Fig. 4). Genes classification related to cellular components were 5 in total, there were cellular anatomical entity (9667 genes), intracellular (4924 genes), protein-containing complex (3650 genes), virion (687 genes) and virion part (687 genes). Molecular functions held 12 classifications of genes among them involving genes in binding (9041 genes), catalytic activity (7027 genes), transporter activity (2115 genes) and structural molecule activity (1027 genes) (Fig. 4).

**Fig. 5:**
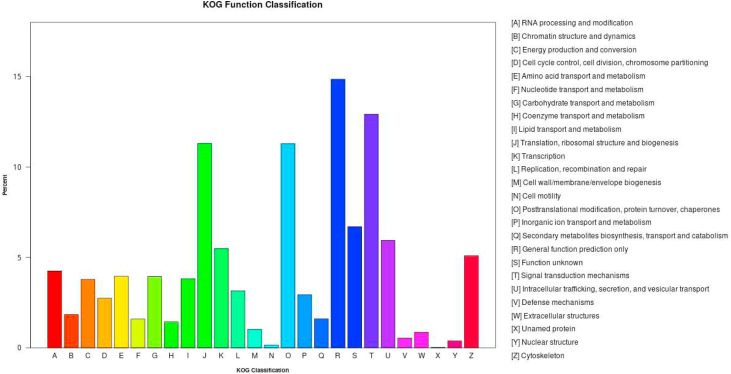
Functional classifications of the assembled unigenes according to the euKaryotic Ortholog Group categories (KOG). X-axis is the names of the 26 KOG group; Y-axis is the percentage of genes annotated under this group in the total annotated genes.

Based on this study a total of 8164 unigenes (17.09%) were annotated in KOG, and these unigenes were categorized into 26 groups of KOG function clusters. Among these, the general function prediction only cluster had the highest number of unigenes (1213), and the signal transduction mechanisms cluster had the second largest number of unigenes (1055 unigenes), followed by translation, ribosomal structure and biogenesis cluster (923 unigenes) (Fig. 5). Next were the posttranslational modification, protein turnover, chaperones cluster (922 unigenes), intracellular trafficking, secretion, and vesicular transport cluster (485 unigenes), transcription cluster (448 unigenes), cytoskeleton cluster (416 unigenes), and RNA processing and modification cluster (347 unigenes). Besides that, the KOG has also annotated about 44 unigenes were classified in defense mechanism clusters (Fig. 5).

**Fig. 6:**
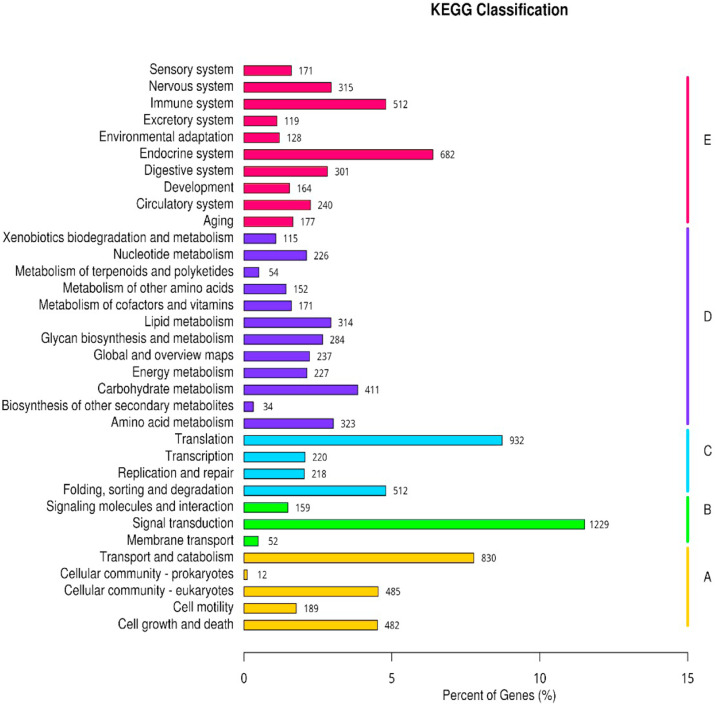
Functional classification of KEGG pathway of assembled unigenes. The KEGG pathways were summarized in five main categories: A, Cellular Processes; B, Environmental Information Processing; C, Genetic Information Processing; D, Metabolism; E, Organismal Systems. The y-axis indicated the name of the KEGG metabolic pathways. The x-axis indicated the percentage of the number of genes annotated under that pathway in the total number of annotated genes.

By using KO annotations, we classified the genes into 32 groups based on their participation in KEGG metabolic pathways (Fig. 6). In this study, among the pathways were signal transduction (1229 unigenes), followed by translation pathways (932 unigenes), transport and catabolism pathways (830 unigenes), endocrine system pathways (682 unigenes) and folding, sorting and degradation pathways (512 unigenes). It has been found that immune system unigenes (512 unigenes) from organismal systems pathways were identified. The immune systems pathway that relates to the KEGG annotation was presented in Table 5. This includes B cell receptor signaling pathway, antigen processing and presentation, chemokine signaling pathway and complement and coagulation. Thus, it was clearly depicted that the conserved immune system pathways were observed from this analysis.

**Table 5:** KEGG pathway immune systems related pathway codes found in *P. vannamei*.

KEGG ID	KEGG pathway involved
KO04612	Antigen processing and presentation
KO04662	B cell receptor signaling pathway
KO04062	Chemokine signaling pathway
KO04610	Complement and coagulation

### Identification and analysis of differential expression genes (DEGs) profile analysis

**Fig. 7:**
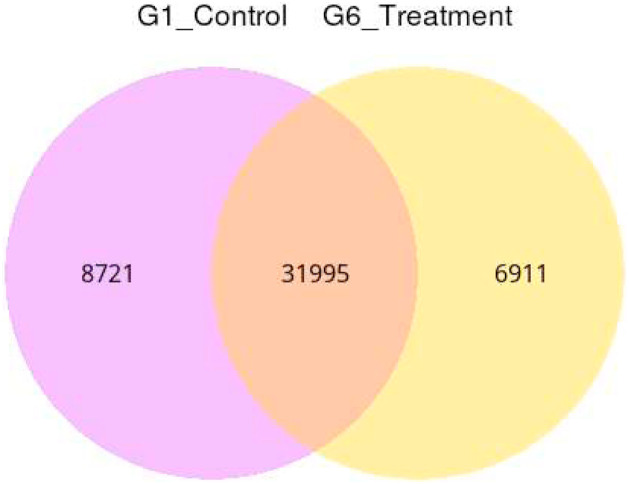
Venn Diagram of Expression Genes. The sum of the numbers in each circle is the total number of genes expressed within a group, and the overlap represents the genes expressed in common between groups. Use Fpkm>0.3 as the criterion.

**Fig. 8:**
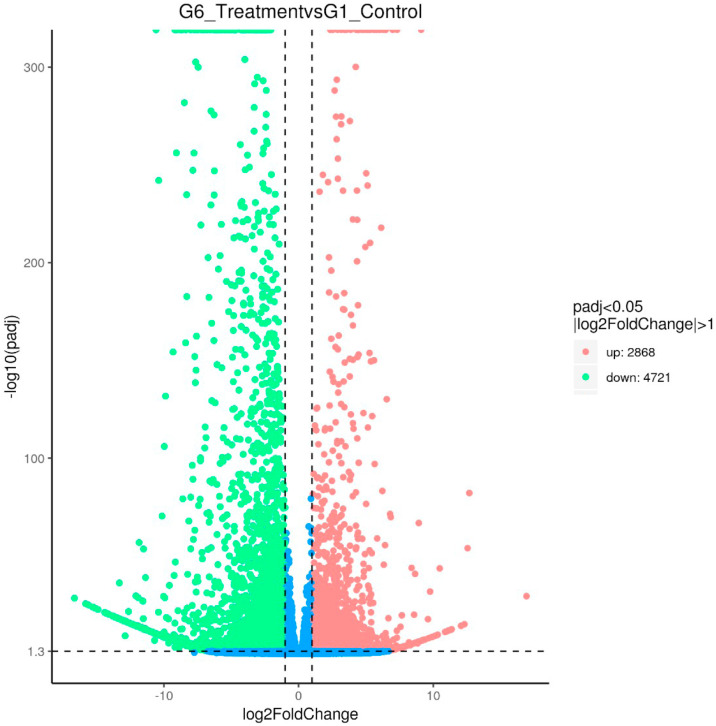
Volcano Plot. The x-axis shows the fold change in gene expression between different samples, and the y-axis shows the statistical significance of the differences. Statistically significant differences are represented by red dots. The sum of the numbers in each circle is the total number of genes expressed within a group, and the overlap represents the genes expressed in common between groups.

Fig. 7 showed the Venn Diagram of Expression Genes. The sum of the numbers in each circle is the total number of genes expressed within a group, and the overlap represents the genes expressed in common between groups. Fig. 8 displayed the results of differentially expressed genes. 2868 genes were up-regulated and 4721 were down-regulated when compared between Group 6 (treatment) and Group 1 (control) taking into account the log_2_FoldChange >1 and padj<0.05 (with biological replicates) (Fig. 8).The read count value from the gene expression level analysis was used as input data to generate the differential expression genes. Volcano plot infers the overall distribution of differential expression genes in Group 1 (control) and Group 6 (treatment) (Fig. 7). The up-regulated difference genes with statistical significance were represented by red dots and the green dots represented the down-regulated difference genes and the blue dots were no difference. Clustering analysis gave the differences and patterns of gene clustering in both Group 1 and Group 6 samples (Fig. 8). Clustering analysis was applied to the common genes of all the differentially expressed genes set and their clusters were calculated. The overall results of FPKM cluster analysis, clustered using the log_10_(FPKM+1) value. Red denotes genes with high expression levels, and green denotes genes with low expression levels. The color range from red to green represents the log_10_(FPKM+1) value from large to small.

### GO enrichment analysis and KEGG pathway enrichment analysis

**Fig. 9:**
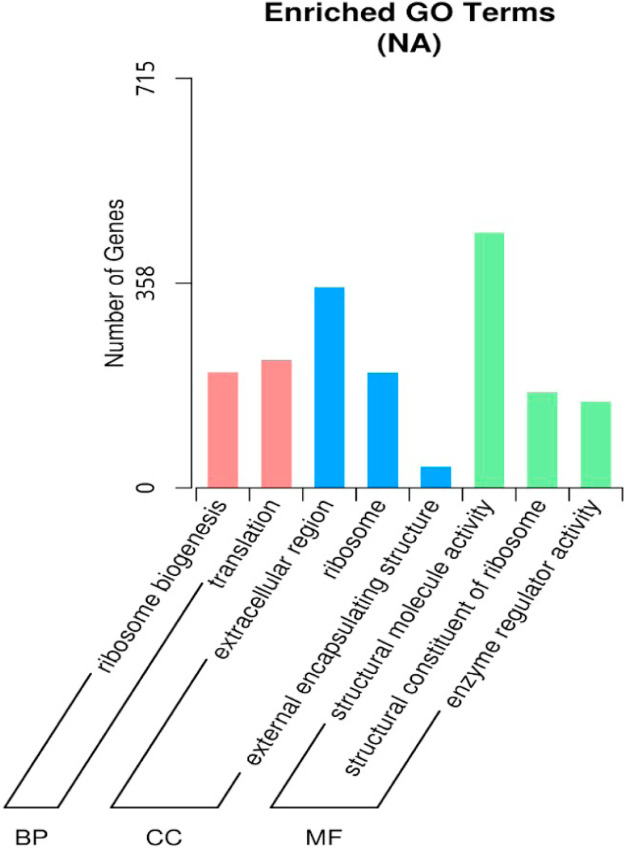
Gene Ontology classifications of the assembled unigenes (GO). The results were categorized into three major categories: cellular component, molecular function, and biological process. The right y-axis indicated the number of genes in a category. The left y-axis indicated the percentage of a specific category of genes in that main category.

**Fig. 10:**
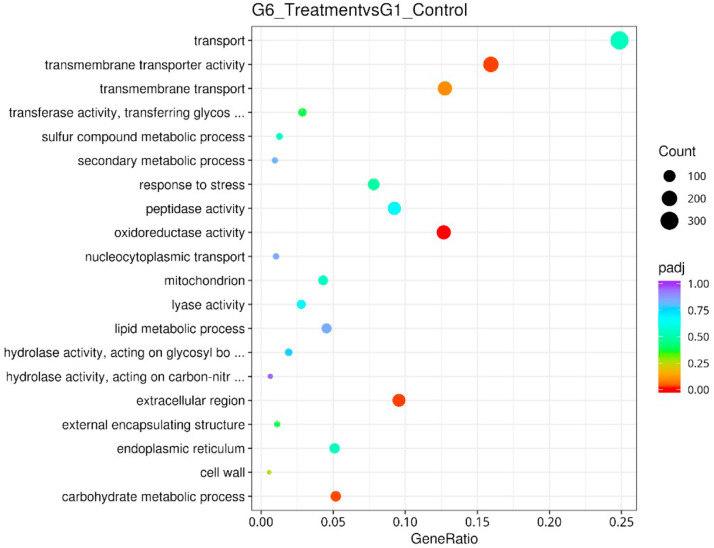
GO enrichment Dot Chart of DEGs. The statistical histogram of up-regulated genes related to GO enrichment (all differential genes).

**Fig. 11:**
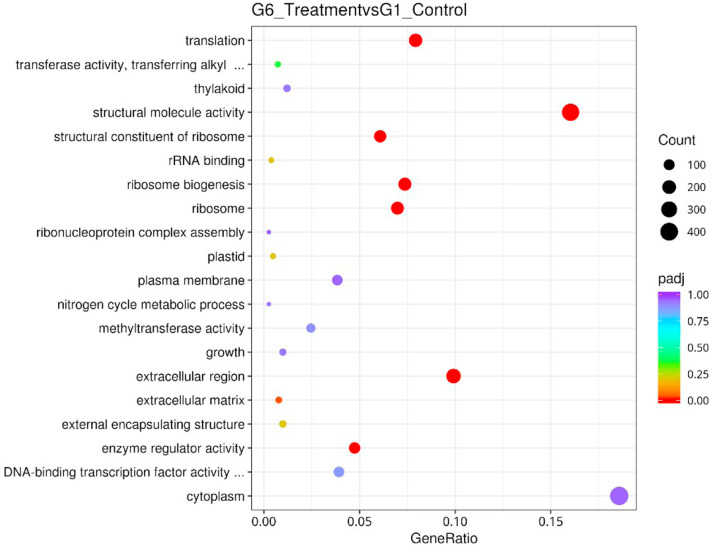
GO enrichment Dot Chart of DEGs. The statistical histogram of down-regulated genes related to GO enrichment (all differential genes).

Fig. 9 showed a GO classifications DEG of the assembled unigenes between Treatment group and Control group. The results were categorized into three major categories which were a cellular component, biological process, and molecular function. Table 6 listed the significantly enriched GO terms in DEGs between the two samples. For cellular components (CC), unigenes clustered under extracellular region, ribosome, and external encapsulating structure were significantly differentially expressed. Meanwhile, for molecular functions (MF), unigenes clustered under structural molecule activity, structural constituent of the ribosome and enzyme regulator were significantly differentially expressed. For biological process, unigenes clustered under ribosome biogenesis and translation were significantly differentially expressed. The number of DEG unigenes up-regulated dan down-regulated under the GO terms were stated in Table 6. Fig. 10 depicted GO enrichment Dot Chart of DEGs for the top up-regulated genes related to GO enrichment (all differential genes) and Fig. 11 GO depicted enrichment Dot Chart of DEGs of the down-regulated genes related to GO enrichment (all differential genes). Among the most upregulated unigenes were transmembrane transporter activity, oxireductase activity, extracellular region and carbohydrate metabolic process (Fig. 10). While, the most downregulated unigenes were transmembrane transporter activity, oxireductase activity, extracellular region and carbohydrate metabolic process structural molecule activity, ribosome biogenesis, translation and extracellular region (Fig. 11).

**Table 6:** Significantly Enriched GO terms in DEGs.

ID	Group	Description	pvalue	padj	Count	Up	Down
GO:0005,198	MF	structural molecule activity	1.46E-34	1.95E-32	446	74	372
GO:0005,576	CC	extracellular region	4.55E-13	3.05E-11	350	120	230
GO:0003,735	MF	structural constituent of ribosome	9.70E-06	0.000433	167	26	141
GO:0030,234	MF	enzyme regulator activity	1.75E-05	0.000587	151	41	110
GO:0042,254	BP	ribosome biogenesis	6.99E-05	0.001874	202	31	171
GO:0005,840	CC	ribosome	0.000111	0.002471	201	39	162
GO:0006,412	BP	translation	0.000309	0.00592	223	39	184
GO:0030,312	CC	external encapsulating structure	0.000808	0.013531	37	14	23

**Fig. 12:**
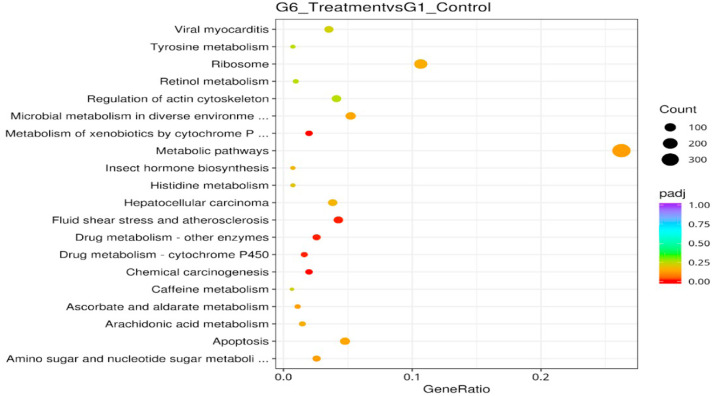
KEGG enrichment scatter plot of DEGs. The y-axis represents the name of the pathway and the x-axis represents the Rich factor. Dot size represents the number of different genes and the color indicates the q-value.

KEGG pathway enrichment analysis of DGE was done to further understand the biological functions of DEGs. The KEGG pathway with corrected P value < 0.05 were considered significantly enriched. A total of 371 DEGs KEGG enrichment pathway was induced following the immunization with the microbial immunostimulants. Based on the results obtained for the DEGs between Group 1 and Group 6, there were six significantly enriched pathways, and the most significantly overrepresented enriched pathways were metabolism of xenobiotics by cytochrome P450 (ID:KO00980), chemical carcinogenesis (ID:KO05204), drug metabolism-other enzymes (ID:KO00983), drug metabolism-cytochrome (KO00982) and fluid shear stress and atherosclerosis (ID:KO05418) (Table 7). The top 20 enriched KEGG pathways corresponding to DEGs detected in both Group 1 and Group 6 were presented as a scatter plot of DEGs in Fig. 12. Among the top enriched KEGG pathways were involving metabolic pathways, ribosome, microbial metabolism in a diverse environment, apoptosis, amino sugar and nucleotide sugar metabolism, hepatocellular carcinoma and chemical carcinogenesis.

**Table 7:** List of DEG-KEGG enrichment pathways following immunization in shrimp, *P. vannamei*.

ID	Description	pvalue	padj	count	up	down
ko00980	Metabolism of xenobiotics by cytochrome P450	1.66E-05	0.00415	27	23	4
ko05204	Chemical carcinogenesis	2.24E-05	0.00415	27	22	5
ko00983	Drug metabolism - other enzymes	9.84E-05	0.010791	35	27	8
ko00982	Drug metabolism - cytochrome P450	0.000132	0.010791	22	18	4
ko05418	Fluid shear stress and atherosclerosis	0.000146	0.010791	58	21	37

### Analysis of immune-related genes and signaling pathways in shrimp, L. *vannamei*

In this study, we also identified differentially expressed immune-related gene such as pathogens-induced interactive response genes, pathogen-associated molecular patterns genes, antimicrobial peptides, proPhenoloxidases system genes, antioxidation genes, phagocytosis-related genes, coagulation system, oxidative stress, proteolytic activity, DSCAM (Down syndrome cell adhesion molecule isoform) and other immune pathway-related genes. Table 8 listed the immune-related DEGs with their annotated identities between (treatment) and Group 1 (control). The unigenes with log_2_(Fold Change)>1 value were considered differentially expressed and were statistically significant based on padj<0.05 (with biological replicates). For pathogens-induced interactive response genes, chitinase (chitinase 1 precursor, partial) and mucin (mucin-5AC-like) were up-regulated in Treatment group compared toc Control group. While for pathogen-associated molecular patterns genes, C-type lectin, von Willebrand factor A domain-containing protein 7, chitin-binding protein and beta-mannosidase were seen up-regulated. Penaeidin, crustin and anti-lipopolysaccharide factor was among the highly expressed antimicrobial gene (Table 8). Prophenoloxidase activating enzyme III, superoxide dismutase and glutathione peroxidase were also among other highly expressed genes involving the proPhenoloxidases system genes and antioxidant genes. The genes involving innate immunity pathways such as phagocytosis-related genes, coagulation system, oxidative stress and proteolytic activity were also found to be up-modulated. The genes involving alternative adaptive immunity called DSCAM (Down syndrome cell adhesion molecule isoform) were also found highly expressed in Treatment Group compared to Control Group. Other immune pathway-related genes such as Wnt16, Wnt2, Wntless protein, toll protein and toll-like receptor involved in Wnt/β-catenin pathway and Toll-like pathways were also significantly overly expressed in Treatment group (Table 8).

**Table 8:** Immune-related DEGs with their annotated identities, annotation and log_2_ (Fold Change) between Group 6 (treatment) and Group 1 (control).

Category/gene ID (cluster)	Annotated homologous identity	Annotation	log_2_ (Fold Change)
***Pathogens-induced interactive response genes***			
4383.0	chitinase	chitinase 1 precursor, partial [Penaeus monodon]	4.6194*
10,528.1411		chitinase 1 precursor [Penaeus vannamei]	4.3622*
23,279.0	Mucin	mucin-5AC-like [Penaeus vannamei]	3.8130*
***Pathogen-associated molecular patterns genes***			
21,901.0	C-type lectin	C-type lectin [Penaeus vannamei]	5.1565*
30,635.0		C-type lectin 5 [Penaeus merguiensis]	4.0125*
5737.0	von Willebrand factor A	von Willebrand factor A domain-containing protein 7-like [Penaeus vannamei]	4.4672*
24,809.0	chitin-binding protein	chitin binding-like protein [Penaeus vannamei]	2.7290*
15,081.0	beta-mannosidase	beta-mannosidase-like isoform X2 [Penaeus vannamei]	1.9231*
***Antimicrobial peptides***			
29,399.1	penaeidin	penaeidin-2b-like [Penaeus vannamei]	9.6574*
8761.0		penaeidin [Penaeus chinensis]	4.5019*
14,151.0	Crustin	crustin 3 [Penaeus vannamei]	4.7437*
10,528.5732		crustin 7 [Penaeus japonicus]	3.5443*
10,528.149	anti-lipopolysaccharide factor	anti-lipopolysaccharide factor isoform 7 [Penaeus monodon]	3.8796*
***proPhenoloxidases system genes***			
23,624.0	prophenoloxidase enzyme	prophenoloxidase activating enzyme III [Penaeus vannamei]	4.4261*
***Antioxidation genes***			
17,848.0	superoxide dismutase	copper/zinc superoxide dismutase isoform 1 [Penaeus japonicus]	3.8191*
21,773.0	glutathione peroxidase	glutathione peroxidase 3 [Penaeus monodon]	2.1917*
			
***Phagocytosis-related genes***			
1865.0	macrophage receptor	macrophage mannose receptor 1-like [Penaeus vannamei]	6.8622*
***Coagulation system***			
645.1	coagulation factor	putative coagulation factor XI [Penaeus vannamei]	7.4852*
13,635.0	clotting enzyme	proclotting enzyme-like [Penaeus vannamei]	1.9422*
***Oxidative stress***			
10,941.0	metallothionein	metallothionein [Penaeus japonicus]	4.6323*
10,528.7746	glutathione S-transferase	glutathione S-transferase Mu 1-like [Penaeus vannamei]	2.7381*
10,528.7689		glutathione S-transferase T2-like [Penaeus vannamei]	2.5746*
10,528.5924	peroxiredoxin	peroxiredoxin [Penaeus monodon]	1.8269*
17,145.0	thioredoxin	thioredoxin-like protein 4A [Centruroides sculpturatus]	1.1732*
***Proteolytic activity***			
2400.1	serine protease inhibitor	serine protease inhibitor 2.1-like [Penaeus vannamei]	7.7263*
8976.0	Trypsin	trypsin-1-like [Penaeus vannamei]	5.1917*
2177.1	lysozyme	i-type lysozyme-like protein 1 [Penaeus vannamei]	4.7909*
15,889.0	carboxypeptidase	carboxypeptidase B-like [Penaeus vannamei]	2.4356*
***DSCAM (Down syndrome cell adhesion molecule isoform)***			
18,316.0	Down syndrome cell adhesion molecule isoform	Down syndrome cell adhesion molecule isoform, partial [Penaeus vannamei]	1.8324*
***Pathway-related genes***			
17,572.0	Wnt/β-Catenin Pathway	Wnt16 [Penaeus vannamei]	1.7684*
10,528.10210		Wnt2 [Penaeus vannamei]	1.7192*
10,528.2836		wntless protein [Penaeus vannamei]	1.2214*
10,528.5394	Toll	toll protein [Penaeus vannamei]	1.6814*
23,589.0		toll-like receptor Tollo [Penaeus vannamei]	1.5753*

The gene with log_2_(Fold Change)>1 value considered differentially expressed and labelled.

⁎ are statistically significant based on padj<0.05 (with biological replicates).

## Discussion

The effect of the immunization has been further elucidated at the transcriptional level to find out its immune response on immunized shrimp’s gene expression compared to control and highlighting the immune-related genes. Based on the differentially expressed genes (DEGs) detected in the KEGG pathway database, several notable changes in the immune-related genes were identified following the immunization of *P. vannamei*. The overall immunological response pattern of the immunized L. *vannamei* during immunization in this study revealed that pathogen-associated molecular patterns (C-type lectin) activated innate immunity and were recognized by pattern recognition receptors (Toll-like receptors) found on host cells [40]. This leads to the activation of signaling pathways (Wnt16, Wnt2) and elevated expression of antimicrobial peptides (anti-lipopolysaccharide factor, penaeidin, crustin), further targeting and killing the invading pathogens [41].

The upregulated mucin, chitinase, and chitin deacetylase gene expressions in immunized shrimp in response suggest an interactive relationship involving chitin [42]. The interaction is important for purposes such as response to bacterial pathogenicity as the bacteria like *V. parahaemolyticus* are capable of utilizing pili (for example, type IV pili) for chitin binding to colonize on the host surface, usually exoskeletons or intestinal cavities [43]. The immunization has also activated transcriptional activities triggering antimicrobial responses to eliminate the invading pathogens. The antimicrobial responses include the activation of proPO system, the release of AMPs, and activation of phagocytosis [44].

The proPO gene expression was upregulated as well to support and sustain the cascade reactions of proPO system. In crustaceans, the prophenoloxidase (proPO) system is an important mechanism of innate immune defense that involves a cascade of serine proteinases converting inactive proPO to active phenoloxidase (PO), thus causing downstream immune actions, such as toll pathway activation, immune gene synthesis, and melanization [45].

The upregulation of antioxidant gene expressions suggested the involvement of first line antioxidant defense [46]. Superoxide dismutase glutathione peroxidase and down syndrome cell adhesion molecule (DSCAM) gene expressions were upregulated indicating the activation of platelet or homologous mechanism and immune memory [44]. In addition to that, the Down syndrome cell adhesion molecule (DSCAM), which is a hypervariable protein that can function as a pathogen-specific recognizing molecule in shrimp was identified to be up-regulated in the immunized shrimp [47]. The up-regulation of this molecule suggested that the inactivated microbial stimulant developed in this study was able to trigger the activation of the alternative adaptive immunity of the shrimp and further enhanced the shrimp immune response against AHPND [47].

The activation of these immune-related signaling pathways was validated by the similarly upregulated AMPs, which include anti-lipopolysaccharide factor (ALF), penaeidin and crustin [47]. The detection of upregulated and pathway-related genes (Wnt/β-Catenin Pathway and Toll-like receptor) indicates the activation of corresponding TLRs, IMD, JAK-STAT, and cytosolic sensing pathways [41]. The currently known shrimp immune signaling pathways vital for disease combating include Janus kinase-Signal transducer and activator of transcription (JAK-STAT) pathway, Immune deficiency (IMD) pathway, TLRs pathway, RNA interference (RNAi) pathway, and P38 mitogen-activated protein kinase (MAPK) pathway [41].

Therefore, this study was able to identify the different types and chronological order of immune response after being immunized by the feed-based inactivated microbial immunostimulant with prebiotic, *Sargassum* sp, and adding notable information to the growing knowledge of immunization in shrimp.Substantial insights have been gained in recent years into important aspects of the crustacean immune system, including the role of phagocytic cells, the prophenoloxidase cascade, melanization system, and antimicrobial peptides [48]. While some of these well-conserved immune effector pathways (such as melanization and antimicrobial peptide production) are reasonably well understood at the biochemical level, the molecular events that underlie the majority of crustaceans such as in shrimp immune reactions remain unknown. Some of the greatest gaps in our knowledge of crustacean immunity concern the molecular basis for its response toward immunostimulation. These findings underscore the importance of a multifaceted approach to managing shrimp health, combining insights from transcriptome analyses and the application of immunostimulants to bolster the shrimp's immune system. By advancing our understanding of the immune responses in L. *vannamei*, we can develop more effective strategies to mitigate the impact of diseases like AHPND, thereby supporting the sustainable growth of the shrimp aquaculture industry. These findings collectively contribute to the development of disease-resistant strategies in shrimp aquaculture, emphasizing the intricate relationship between the shrimp immune system, transcriptomic responses, and immunostimulation for enhancing the health and resilience of L. *vannamei* aquaculture product.

Nevertheless, this study acknowledges the absence of qRT-PCR validation as a limitation of the current work. Future studies will focus on targeted qRT-PCR validation of selected immune-related genes to further substantiate the transcriptomic findings and strengthen the reliability of gene expression analysis.

## Conclusion

Following immunization of L. *vannamei* with feed-based inactivated microbial cells and *Sargassum* sp. as immunostimulants, several notable changes in immune-related genes were discovered based on differentially expressed genes (DEGs) revealed in the KEGG pathway database. The dominant immune pathways involved in the observed modulation are primarily the innate immune signaling pathways, particularly the Toll-like receptor (TLR), immune deficiency (IMD), and JAK–STAT pathways, supported by the upregulation of pattern recognition receptors and key signaling genes. In addition, the prophenoloxidase (proPO) system and antimicrobial peptide responses (e.g., penaeidin, crustin, and ALF) were strongly activated, indicating that these pathways collectively play a central role in mediating the immunostimulatory effects observed in L. *vannamei*. Aside from that, our study was able to determine the various kinds and chronological sequence of immune response after being immunised with the inactivated microbial immunostimulant, contributing significantly to the developing understanding of immunisation in shrimp. These findings help to develop disease-resistant strategies in shrimp aquaculture by emphasizing the complex relationship between the shrimp immune system, transcriptomic responses, and immunostimulation for improving the health and resilience of L. *vannamei* aquaculture productions.

## CRediT authorship contribution statement

**M.A. Amatul-Samahah:** Writing – original draft, Methodology, Investigation, Funding acquisition, Formal analysis. **F.M.I. Natrah:** Methodology, Investigation, Supervision. **M.N.A. Amal:** Methodology, Investigation, Supervision. **M.Y. Ina-Salwany:** Writing – review & editing, Visualization, Supervision, Resources, Project administration, Funding acquisition, Conceptualization.

## Declaration of competing interest

The authors declare that they have no known competing financial interests or personal relationships that could have appeared to influence the work reported in this paper.

## Data Availability

Data will be made available on request.

## References

[1] Muahiddah N., Rangga I., Affandi W., Ayu D. (2022). The effect of immunostimulants from natural ingredients on vanamei shrimp (*Litopenaeus vannamei*) in increasing non-specific immunity to fight disease. J. Fish Health.

[2] Kumar S., Verma A.K., Singh S.P. (2023). Immunostimulants for shrimp aquaculture: paving pathway towards shrimp sustainability. Environ. Sci. Pollut. Res..

[3] Kilawati Y., Islamy R.A. (2021). Immunostimulant activity of *Gracilaria* sp. and *Padina* sp. on immune system of Vannamei shrimp (*Litopenaeus vannamei*) against Vibrio harveyi. J. Aquacult. Fish Health.

[4] Amatul-Samahah M.A., Muthukrishnan S., Al-saari N., Ikhsan N.F.M., Amal M.N.A., Zamri-Saad M., Yusof M.T., Ina-Salwany M.Y., Tanaka M., Mino S., Sawabe T. (2022). Genome sequence of *vibrio parahaemolyticus* C5A causing acute hepatopancreatic necrosis disease in shrimp isolated from Malaysia shrimp pond culture. Gene Rep..

[5] Dong X., Bi D., Wang H., Zou P., Xie G., Wan X., Yang Q., Zhu Y., Chen M., Guo C., Liu Z., Wang W., Huang J. (2017). pirABvp-bearing *V. parahaemolyticus* and *V. campbelli* pathogens isolated from the same AHPND-affected pond possess high similar pathogenic plasmids. Front. Microbiol..

[6] Haifa-Haryani W.O., Azzam-Sayuti M., Amatul-Samahah M.A., Chin Y.K., Zamri-Saad M., Anas S., Natrah I., Amal M.N.A., Ina-Salwany M.Y. (2023). Pathogenesis and virulence factors of *vibrio* spp. Isolated from cultured shrimp in Peninsular Malaysia. Aquac. Res..

[7] Miao M., Shi-he L., Yuan L., Yang Y., Fuhua L. (2023). Transcriptome analysis on hepatopancreas reveals the metabolic dysregulation caused by *Vibrio parahaemolyticus* Infection in *Litopenaeus vannamei*. Biology. (Basel).

[8] Tran L., Nunan L., Redman R.M., Mohney L.L., Pantoja C.R., Fitzsimmons K., Lightner D.V. (2013). Determination of the infectious nature of the agent of acute hepatopancreatic necrosis syndrome affecting penaeid shrimp. Disease Aqua. Organ..

[9] Anirudhan A., Iryani M.T.M., Andriani Y., Sorgeloos P., Tan M.P., Wong L.L., Mok W.J., Ming W., Yantao L., Lau C.C., Sung Y.Y. (2023). The effects of *Pandanus tectorius* leaf extract on the resistance of White-leg shrimp *Penaeus vannamei* towards pathogenic *Vibrio parahaemolyticus*. Fish Shellfish Immunol..

[10] Huang Z., Zhang Y., Zheng X., Liu Z., Yao D., Zhao Y., Chen X., Aweya J.J. (2022). Functional characterization of arginine metabolic pathway enzymes in the antibacterial immune response of penaeid shrimp. Developm. Comparat. Immunol..

[11] Robalino J., Carnegie R.B., O'Leary N., Ouvry-Patat S.A., de la Vega E., Prior S., Gross P.S., Browdy C.L., Chapman R.W., Schey K.L., Warr G. (2009). Contributions of functional genomics and proteomics to the study of immune responses in the Pacific white leg shrimp *Litopenaeus vannamei*. Vet. Immunol. Immunopathol..

[12] Arayamethakorn S., Uengwetwanit T., Karoonuthaisiri N., Methacanon P., Rungrassamee W. (2023). Comparative effects of different bacterial lipopolysaccharides on modulation of immune levels to improve survival of the black tiger shrimp. J. Invertebr. Pathol..

[13] Ren X., Yu Z., Xu Y., Zhang Y., Mu C., Liu P., Li J. (2020). Integrated transcriptomic and metabolomic responses in the hepatopancreas of kuruma shrimp (*Marsupenaeus japonicus*) under cold stress. Ecotoxicol. Environ. Saf..

[14] Qin Z., Babu V.S., Wan Q., Zhou M., Liang R., Muhammad A., Zhao L., Li J., Lan J., Lin L. (2018). Transcriptome analysis of Pacific white shrimp (*L. vannamei*) challenged by *V. parahaemolyticus* reveals unique immune-related genes. Fish Shellfish Immunol..

[15] Senghoi W., Thongsoi R., Yu X.Q., Runsaeng P., Utarabhand P. (2019). A unique lectin composing of fibrinogen-like domain from *Fenneropenaeus merguiensis* contributed in shrimp immune defense and firstly found to mediate encapsulation. Fish Shellfish Immunol..

[16] Wang Z., Zhang Y., Aweya J.J., Lin Z., Yao D., Zheng Z. (2023). The histidine phosphatase LHPP of *Penaeus vannamei* is involved in shrimp hemocytes apoptosis. Fish. Shellfish. Immunol. Rep..

[17] Campa-Cordova A.I., Hernandez-Saavedra N.Y., Philippis R.D, Ascencio F. (2002). Generation of superoxide anion and SOD activity in haemocytes and muscle of American white shrimp (*L. vannamei*) as a response to β-glucan and sulphated polysaccharide. Fish Shellfish Immunol..

[18] Kumar S., Sunagar R., Gosselin E. (2019). Bacterial protein toll-like-receptor agonists: a novel perspective on vaccine adjuvants. Front. Immunol..

[19] Martin G.G., Graves B. (2005). Fine structure and classification of shrimp haemocytes. J. Morphol..

[20] Zhang Z.F., Shao M., Ho-Kang K. (2006). Classification of haematopoietic cells and haemocytes in Chinese prawn *F. chinensis*. Fish Shellfish Immunol..

[21] Azad I.S., Panigrahia A., Gopala C., Paulpandia S., Mahimaa C., Ravichandrana P. (2006). Routes of immunostimulation vis-a-vis survival and growth of *P. monodon* postlarvae. Aquaculture.

[22] Amatul-Samahah M.A., Muthukrishnan S., Omar W.H.H.W., Ikhsan N.F.M., Ina-Salwany M.Y. (2020). *Vibrio* spp. Associated with acute hepatopancreatic necrosis disease (AHPND) found in penaeid shrimp pond from east cost of peninsular Malaysia. J. Environ. Biol..

[23] Patil P.K., Gopal C., Panigrahi A., Rajababu D., Pillai S.M. (2014). Oral administration of formalin killed *V. anguillarum* cells improves growth and protection against challenge with *Vibrio harveyi* in banana shrimp. Lett. Appl. Mirobiol..

[24] Nazaruddin M.F., Yusoff F., Idrus E.Z., Aliyu-Paiko M. (2020). Brown seaweed *sargassum polycystum* as dietary supplement exhibits prebiotic potentials in Asian sea bass *Lates calcarifer* fingerlings. Aquac. Rep..

[25] Ray A.K., Gopal C., Solanki H.G., Ravisankar T., Patil P.K. (2017). Effect of orally administered Vibrio bacterin on immunity, survival and growth in tiger shrimp (*P. monodon*) grow out culture ponds. Lett. Appl. Microbiol..

[26] Manan H., Moh J.H.Z., Othman F., Ikhwanuddin M. (2015). Histopathology of the Hepatopancreas of Pacific White Shrimp, *Penaeus vannamei* from none early mortality syndrome (EMS) shrimp ponds. J. Fish Aquat. Sci..

[27] Rio D.C., Ares M., Hannon G.J., Nilsen T.W (2010). Purification of RNA using TRIzol (TRI reagent). Cold Spring Harb Protocols.

[28] Kukurba K.R., Montgomery S.B. (2015). RNA sequencing and snalysis. Cold Spring Harbour Protocols.

[29] Grabherr M., Haas B., Yassour M. (2011). Full-length transcriptome assembly from RNA-seq data without a reference genome. Nat. Biotechnol..

[30] Davidson N.M., Oshlack A. (2014). Corset: enabling differential gene expression analysis for de novo assembled transcriptomes. Genome Biol..

[31] Simão F.A., Waterhouse R.M., Ioannidis P., Kriventseva E.V., Zdobnov E.M. (2015). BUSCO: assessing genome assembly and annotation completeness with single-copy orthologs. Bioinformatics.

[32] Buchfink B., Xie C., Huson D.H. (2015). Fast and sensitive protein alignment using DIAMOND. Nat. Methods.

[33] Eddy S.R. (2011). Accelerated profile HMM searches. PLoS. Comput. Biol..

[34] Götz S., García-Gómez J.M., Terol J., Williams T.D., Nagaraj S.H., Nueda M.J., Robles M., Talón M., Dopazo J., Conesa A. (2008). High-throughput functional annotation and data mining with the Blast2GO suite. Nucleic. Acids. Res..

[35] Moriya Y., Itoh M., Okuda S., Yoshizawa A., Kanehisa M. (2007). KAAS: an automatic genome annotation and pathway reconstruction server. Nucleic. Acids. Res..

[36] Anders S., Huber W. (2010). Differential expression analysis for sequence count data. Genome Biol..

[37] Robinson M.D., McCarthy D.J., Smyth G.K. (2010). edgeR: a bioconductor package for differential expression analysis of digital gene expression data. Bioinformatics.

[38] Mao X., Cai T., Olyarchuk J.G., Wei L. (2005). Automated genome annotation and pathway identification using the KEGG Orthology (KO) as a controlled vocabulary. Bioinformatics.

[39] Kanehisa M., Araki M., Goto S., Hattori M., Hirakawa M., Itoh M., Katayama T., Kawashima S., Okuda S., Tokimatsu T., Yamanishi Y. (2008). KEGG for linking genomes to life and the environment. Nucleic. Acids. Res..

[40] Wang X.W., Wang J.X. (2013). Pattern recognition receptors acting in innate immune system of shrimp against pathogen infections. Fish Shellfish Immunol..

[41] Chen Y., Li X., He J. (2014). Recent advances in researches on shrimp immune pathway involved in white spot syndrome virus genes regulation. J. Aquac. Res. Dev..

[42] Kondo T., Kawai T., Akira S. (2012). Dissecting negative regulation of toll-like receptor signaling. Trends. Immunol..

[43] Pruzzo C., Vezzulli L., Colwell R.R. (2008). Global impact of *Vibrio cholerae* interactions with chitin. Environ. Microbiol..

[44] Li C., Wang S., He J. (2019). The two NF-κb pathways regulating bacterial and WSSV infection of shrimp. Front. Immunol..

[45] Rao R., Bhassu S., Zhu Y.B.R., Alinejad T., Hassan S.S., Wang J. (2016). A transcriptome study on *M. rosenbergii* hepatopancreas experimentally challenged with white spot syndrome virus (WSSV). J. Invertebr. Pathol..

[46] Chien C., Lin T., Chi C., Liu C. (2020). Probiotic, Bacillus subtilis E20 alters the immunity of white shrimp, Litopenaeus vannamei via glutamine metabolism and hexosamine biosynthetic pathway. Fish Shellfish Immunol..

[47] Chang Y.H., Devdas R., Ng T.H., Wang H.C. (2018). What vaccination studies tell us about immunological memory within the innate immune system of cultured shrimp and crayfish. Developm. Comparat. Immunol..

[48] Kumar V., Roy S., Behera B.K., Bossier P., Das B.K. (2021). Acute hepatopancreatic necrosis disease (AHPND): virulence, pathogenesis and mitigation strategies in shrimp aquaculture. Toxins. (Basel).

